# Pediatric Pott's Disease of the Sacrum: A Case Report

**DOI:** 10.1002/ccr3.70807

**Published:** 2025-08-21

**Authors:** Abdul Mannan, Zainab Jahangir, Muhammad Owais, Muhannad Bushra Masaad Ahmed

**Affiliations:** ^1^ Pakistan Institute of Medical Sciences Islamabad Pakistan; ^2^ Rawalpindi Institute of Cardiology Rawalpindi Pakistan; ^3^ Liaquat Universty of Medical & Health Sciences Jamshoro Pakistan; ^4^ Faculty of Medicine and Health Sciences University of Gadarif Gadarif Sudan

**Keywords:** pediatrics, Pott's disease, sacrum, tuberculosis, tuberculous spondylitis

## Abstract

Isolated sacral tuberculosis in children is rare and can present without typical signs of inflammation. Early recognition and initiation of antituberculosis therapy are crucial to prevent complications such as bone destruction, deformity, and paraplegia.

**Abbreviations**
AFB: acid‐fast bacilli (mentioned indirectly via Ziehl‐Neelsen staining)
ATT: antituberculosis therapy
BCG: bacille Calmette–Guérin
CRP: C‐reactive protein
EPI: expanded programme on immunization
ESR: erythrocyte sedimentation rate
HIV: human immunodeficiency virus
MRI: magnetic resonance imaging
MTB/RIF:
*Mycobacterium tuberculosis* /resistance to rifampicin
PCR: polymerase chain reaction
TB: tuberculosis

## 1 Introduction

Skeletal tuberculosis (TB) accounts for 10%–25% of all extrapulmonary TB cases globally, posing a significant diagnostic challenge due to its nonspecific presentation [1]. Among these, “Pott's disease,” also known as tuberculous spondylitis, is the most common form, making up nearly half of all bone and joint TB cases [2]. Common signs and symptoms of skeletal TB include back pain, localized tenderness, swelling, and neurological deficits in some cases. However, sacral involvement often presents atypically, making early diagnosis challenging. The vast majority of spinal TB cases (95%) occur in the lower thoracic and upper lumbar regions of the spine, while about 5% are seen in the cervical region [3, 4, 5, 6, 7]. Sacral involvement in TB is extremely rare, even in large case series, where no isolated sacral TB cases were identified [3, 4, 5, 6, 7]. Children, in particular, are at risk of developing spinal deformities such as kyphosis, which can lead to significant mobility issues due to their immature and flexible spines [8, 9]. The diagnosis of extrapulmonary TB remains challenging due to its nonspecific clinical presentation, despite the growing availability of advanced imaging techniques [10, 11, 12, 13, 14, 15]. Here, we present a rare case of sacral TB in a 3‐year‐old child, contributing to the limited literature on this unusual presentation of pediatric Pott's disease.

## 2 Case History/Examination

A 3‐year‐old boy presented to our tertiary care hospital's outpatient department with the chief complaint of inability to walk for 2 months. Prior to the onset of symptoms, he had been in his usual state of health, with an uneventful developmental and nutritional history. He was the second child of a non‐consanguineous marriage from a low socioeconomic background. There was no history of contact with TB patients or adults with chronic cough, and no risk factors such as sickle cell disease, diabetes, or immunosuppressive conditions were identified. The child had also been fully vaccinated according to the expanded programme on immunization (EPI) schedule.

## 3 Methods

### 3.1 Differential Diagnosis and Investigations

On general examination, the child was afebrile and showed mild anemia. His growth parameters were within normal ranges: height (75th percentile), weight (25th percentile), and orbitofrontal circumference (75th percentile). The bacille Calmette–Guérin (BCG) scar was absent, and systemic examination of the chest and abdomen revealed no abnormalities. Neurological evaluation indicated reduced power (3/5) in the lower limbs, with brisk deep tendon reflexes, but no sensory deficits were observed. Routine laboratory tests, including a total leukocyte count of 9.72 × 10^9^/L, neutrophils at 47.9%, lymphocytes at 43.0%, hemoglobin at 10.3 g/dL, Erythrocyte Sedimentation Rate (ESR) at 123 mm/h, and C‐reactive protein (CRP) at 71.3 mg/L, suggested an inflammatory process. Hepatitis B surface antigen and human immunodeficiency virus (HIV) markers were negative, and renal and liver function tests were normal. A chest X‐ray revealed no signs of pulmonary TB. A lateral view X‐ray of the spine showed a presacral mass, leading to a preliminary diagnosis of spinal mass, possibly due to a spinal tumor or TB.

**FIGURE 1:**
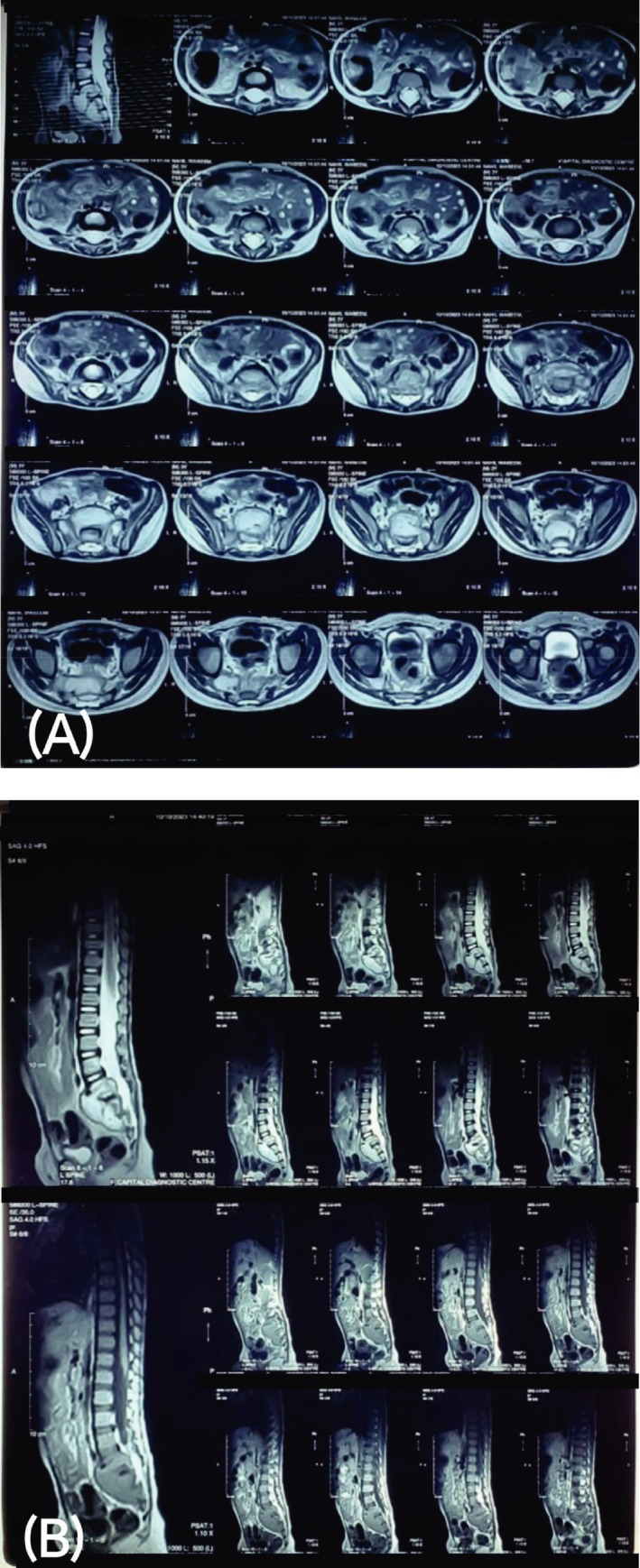
(A, B) Multi‐planar, multi‐sequence, non‐contrast enhanced magnetic resonance imaging of the lumbar spine showing a cold abscess in the pre‐sacral region of S1–S2 with intervertebral disc involvement. (A) Axial T2‐weighted MR images reveal a well‐defined, hyperintense lesion anterior to the S1–S2 vertebral bodies, consistent with a fluid‐containing cold abscess in the presacral space. The lesion causes anterior displacement of adjacent pelvic structures. The surrounding vertebral bodies show altered marrow signal intensity, and there is loss of normal disc height and signal at S1–S2, indicating discitis. (B) Sagittal and coronal T2‐weighted MR images confirm the presence of the presacral abscess tracking along the anterior aspect of the sacrum. The lesion appears hyperintense on T2WI and hypointense on T1WI, with irregular margins. The S1–S2 intervertebral disc shows signs of involvement, including reduced disc height and signal intensity, suggesting disc space infection (discitis). Vertebral endplate irregularity and bone marrow edema are also noted, which are consistent with spondylodiscitis.

Given the child's atypical presentation, the differential diagnoses included both spinal tumors and TB. Further imaging, including an MRI of the lumbar spine, revealed altered signals in the presacral space and destruction of the endplates at S1 and S2 (See Figure 1A,B). These findings were highly suggestive of a cold abscess typical of spinal TB. An ultrasound‐guided pus aspiration from the cold abscess was performed by an interventional radiologist, and subsequent analysis of the aspirated fluid was done. Although Ziehl–Neelsen staining did not reveal acid‐fast bacilli, a polymerase chain reaction (PCR) using the GenXpert MTB/RIF ( *Mycobacterium tuberculosis* /resistance to rifampicin) confirmed the presence of *M. tuberculosis* , thus diagnosing the child with spinal TB.

## 4 Treatment

The child was started on a four‐drug antituberculosis therapy (ATT) regimen: Isoniazid (10 mg/kg/day), Rifampicin (15 mg/kg/day), Pyrazinamide (30 mg/kg/day), and Ethambutol (20 mg/kg/day). This treatment was prescribed for 9 months, in line with standard protocols followed by our department. Surgical intervention was not deemed necessary.

## 5 Conclusion and Results

The child showed remarkable recovery over the treatment period. By the one‐year follow‐up, the patient was symptom‐free and had regained the ability to walk. Hemoglobin levels had improved to 11.1 g/dL, and erythrocyte sedimentation rate (ESR) had normalized to 13 mm/h. No follow‐up imaging was performed due to the family's financial constraints, but the child remained in good health.

Histopathological examination was not conducted due to resource limitations. Future cases would benefit from obtaining biopsy samples to confirm diagnosis and rule out malignancies.

To monitor progress in the absence of imaging, the patient underwent regular physical exams to assess motor function recovery. Blood tests, including ESR and CRP, were used to evaluate inflammation levels.

## 6 Discussion

Sacrum Pott's disease is less prevalent than cervical, thoracic, and lumbar region TB. Spinal TB is typically diagnosed in young adults and older patients but is rarely seen in children [15] in a literature review of isolated sacral TB, the age of affected individuals ranges from 5 to 73 years with a mean age of 30 years [16], while in our study a child presented at an age < 5 years.

Early diagnosis of sacral TB remains a challenge due to its rarity and nonspecific presentation. In this case, the delay in considering TB as a differential resulted from the absence of typical systemic signs. Earlier imaging and heightened suspicion in endemic areas could potentially improve outcomes by initiating timely treatment.

The symptoms of TB in the sacrum can vary widely; although back pain and localized tenderness are typical signs of TB in the spine, some patients experience spinal deformity and neurological problems without these usual symptoms [17, 18, 19]. Our patient also presented in an atypical manner with kyphosis and absence of signs of inflammation. Further, kyphosis occurs more commonly in children when there is thoracic spine involvement [20], while in our case even involvement of the sacrum caused the posture to be abnormal along with an inability to walk properly.

In our patient, the BCG scar mark was absent despite completely following the EPI vaccination schedule; thus, this indicated an incomplete immune response, as scarring at the BCG vaccination site, appearing in 65% to 100% of infants, is considered a substitute measure for immune response [21]. This finding helped us consider spinal TB as an important differential diagnosis in our patient.

The diagnostic process thoroughly evaluated malignancies, including chordomas, which frequently mimic TB in this anatomical region. Distinguishing imaging features, such as the presence of a cold abscess and the lack of typical chordoma calcifications, alongside PCR confirmation of *Mycobacterium tuberculosis* , solidified the diagnosis [16]. Distinguishing features included imaging findings of a cold abscess and PCR confirmation of *M. tuberculosis* . Additionally, the absence of mass effect or typical chordoma calcifications supported the diagnosis of TB.

Diagnosing Pott's disease is difficult due to its gradual and unpredictable onset, often with subtle or nonspecific symptoms and a lack of conclusive laboratory tests. Consistent with the study by Azbaoui et al. in our case, standard microbiological tests come back negative due to low bacterial loads in extrapulmonary TB like tuberculous spondylitis [15]; thus, further radiological investigations are required. Furthermore, skeletal TB can closely resemble bone cancer like chordoma, osteoclastoma, and giant cell tumor, which often leads to misdiagnosis, delaying the start of appropriate treatment. Thus, even when imaging and clinical signs strongly point towards extrapulmonary TB, it can be hard to rule out malignancy based on imaging alone [10]. Therefore, a definitive diagnosis requires confirmation through histopathological examination and bacteriological testing with molecular biological techniques of surgical biopsies of suspicious lesions, as in our case.

The standard treatment for this condition typically involves a four‐drug regimen of rifampicine, isoniazide, pyrazinamide, and ethambutol for the first 2 months, followed by a continuation phase with two drugs. There is some debate in the medical community about the ideal duration of treatment, with recommendations ranging from 6 to 18 months [1, 22]. In this particular case, the patient received a total of 9 months of antituberculous therapy and showed positive progress. In conclusion, sacral TB occurring in isolation in the pediatric population is a rare occurrence; however, a heightened sense of suspicion is imperative in cases where unusual clinical and radiological characteristics of a sacral lesion are observed, especially in endemic regions to prevent bone destruction, deformity, and paraplegia in children.

## Author Contributions

**Abdul Mannan:** supervision, writing – original draft, writing – review and editing. **Zainab Jahangir:** software, writing – original draft, writing – review and editing. **Muhammad Owais:** writing – original draft, writing – review and editing. **Muhannad Bushra Masaad Ahmed:** writing – original draft, writing – review and editing.

## Consent

Written informed consent was obtained from the patient's parent/legal guardian for publication of this case report and any accompanying images. All efforts were made to ensure patient anonymity and confidentiality in accordance with ethical guidelines.

## Conflicts of Interest

The authors declare no conflicts of interest.

## Data Availability

The data that support the findings of this study are available on request from the corresponding author. The data are not publicly available due to privacy or ethical restrictions.
